# Using qualitative and participatory methods to refine implementation strategies: universal family psychosocial screening in pediatric cancer

**DOI:** 10.1186/s43058-021-00163-4

**Published:** 2021-06-05

**Authors:** Janet A. Deatrick, Anne E. Kazak, Rebecca E. Madden, Glynnis A. McDonnell, Katherine Okonak, Michele A. Scialla, Lamia P. Barakat

**Affiliations:** 1 Department of Family and Community Health, University of Pennsylvania School of Nursing, Philadelphia, USA; 2 ABPP Center for Healthcare Delivery Science, Nemours Pediatric Healthcare System, Rockland Center One, 1701 Rockland Road, Wilmington, USA; 3 Department of Pediatrics, Sidney Kimmel Medical School of Thomas Jefferson University, Philadelphia, USA; 4 Divison of Oncology, The Children’s Hospital of Philadelphia, Philadelphia, USA; 5 Department of Pediatrics, Perelman/School of Medicine of the University of Pennsylvania, Philadelphia, USA

**Keywords:** Health-care delivery, Health-care providers, Implementation, Pediatric, Cancer, Psychosocial Assessment Tool © (PAT), Risk screening, Health equity, Oncology

## Abstract

**Background:**

Children with cancer and their families are at risk for short- and long-term psychosocial difficulties. Screening for psychosocial risk remains inconsistent, leading to inequitable access to psychosocial services. The Psychosocial Assessment Tool (PAT) is an evidence-based caregiver report screener of family psychosocial risk ready for implementation in a nationwide cluster randomized trial that will test two implementation strategies across 18 pediatric cancer centers. The current study, conducted in preparation for the trial, solicited the perspectives of key stakeholders about two proposed implementation strategies identified during previous research which focus on health equity and screening of all families (universal screening). Results were used to refine the implementation strategies for testing in the subsequent trial.

**Methods:**

Semi-structured interviews with 19 key stakeholders (parents, health care providers, pediatric oncology organizations, and pediatric healthcare leaders) were conducted regarding the two implementation strategies. Strategy I is a training webinar; Strategy II is training + implementation enhanced resources, which includes a champion at each site and monthly peer support consultation calls. Data were analyzed using directed content analysis with deductively derived codes based on the Interactive Systems Framework and inductive codes based on emerging data.

**Results:**

Stakeholder interviews provided rich data to rigorously modify the proposed implementation strategies. Implementation strategies were modified in consistent with these recommendations: engaging providers by framing family psychosocial screening as an opportunity for more efficient and effective practice; setting clear expectations about the importance of screening 100% of children and their families to achieve the goal of universal screening, equity of care, and reduction of disparities; and adapting successful strategies for systematic implementation of screening to ensure optimal engagement with children and their families throughout their care.

**Conclusions:**

Stakeholder input strengthened implementation strategies by suggesting modifications that emphasized health equity and reduction in health disparities. Using implementation science methods to build on a long-standing program of research provided practical insights about immediate needs of families and historical insights regarding structural inequities such as language differences and access to services. Resulting strategies address all levels of the social ecology for children’s cancer care, including the patient, family, provider, healthcare system, and community.

**Trial registration:**

NCT04446728 June 23, 2020

**Supplementary Information:**

The online version contains supplementary material available at 10.1186/s43058-021-00163-4.

Contributions to the literature

- Filled gap in the literature related to the process of modifying implementation strategies derived from a long-standing program of research focused on health equity.
- Mapped qualitative themes and proposed implementation strategies, modifications, and links with theory.
- Described recommendations for implementation strategies to promote health equity and to reduce health disparities.

## Background

The Institute of Medicine and the Standards of Psychosocial Care in Pediatric Cancer recognize the importance of psychosocial care to achieve equitable psychosocial and health outcomes for children with cancer and their families [1–3]. The first standard states that youth with cancer and their families should routinely receive systematic psychosocial screening to guide a targeted provision of care to reduce distress, bolster resources, and decrease health inequities [4]. While most pediatric cancer clinics have at least minimal psychosocial staffing, screening is not consistently available [5, 6].

**Fig. 1:**
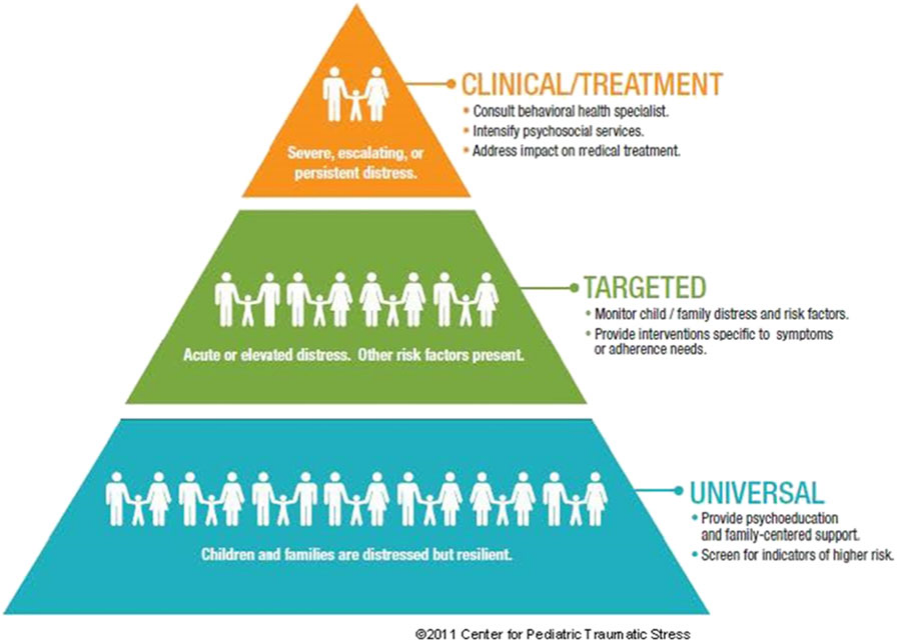
Pediatric psychosocial preventative health model

The Psychosocial Assessment Tool Version 3 (PAT), an all literacy, evidence-based, caregiver-report, family psychosocial risk screener, is validated in English and Spanish and ready for implementation. The PAT is completed using a branching, web-based application in about 10 min. A total score and seven subscale (e.g., social support, child problems) scores are automatically generated. The PAT total score is based on the number of endorsed, high-risk items and maps on to the three-tier Psychosocial Preventative Health Model (PPPHM) (See Fig. 1) [7–9], representing the distribution of psychosocial risks, resources, and adaptation across the population of families from universal (low) and targeted (moderate) to clinical (severe) risk levels.

By screening and identifying specific needs early in the treatment process for all families, the PAT facilitates equitable psychosocial care matched to family needs, family and staff communication, and quality medical care [10]. In a study comparing the use of the PAT in psychosocial screening at diagnosis of a child with cancer with usual care, those screened with the PAT had more psychosocial risks documented in the medical record and social work notes and received more services [11]. Reductions in maternal anxiety and depression have been associated with sharing PAT screening results with staff [12].

The PAT is used in 29% of pediatric cancer programs in the USA [13] and 50 international sites; however, information about the universality and sustainability of the systematic family psychosocial screening with the PAT in practice is unknown [13]. In a multicenter pilot study in 12 cancer centers testing implementation strategies consisting of a workshop and consultation calls, 75% of study sites adopted the PAT. Data gathered via the PAT Implementation Questionnaire about challenges to implementation included staff time required to administer the PAT, methods to communicate results, resources to address identified needs, and skills to address language barriers and cultural considerations [14].

To advance the goal of universal family psychosocial risk screening in pediatric cancer centers and reduce health inequities, we used the Interactive Systems Framework for Dissemination and Implementation (ISF) [15]. The ISF describes three steps (systems) needed to bridge the gap between science and practice by understanding the barriers and resources of the different systems to consider when optimizing implementation strategies [16]. Our broader program of research on the implementation of the PAT utilizes all three steps of the ISF. This paper describes the first step (Prevention Synthesis and Translation) in which the perspectives of key stakeholders were gathered to refine the two implementation strategies to enhance their effectiveness for testing in the subsequent implementation trial. In the second step (Prevention Support System of the ISF), a cluster randomized implementation trial in 18 pediatric cancer programs compares two PAT implementation strategies selected based on prior research and on the Expert Recommendations for Implementing Change (ERIC) [15–17]. Strategy I is a training webinar that addresses family psychosocial adjustment in pediatric cancer, pediatric psychosocial standards of care, the PPPHM Model, description of the PAT, details of using the PAT, and development of an Implementation Plan for using the PAT. Strategy II included Training + Implementation Expanded Resources (TIER). Training is combined with group consultation calls to foster problem-solving and peer support about issues encountered during implementation and an internal champion to support and advocate for screening and assisting with problem solving about screening. In the third step (Prevention Delivery System of the ISF), which implements the innovation into practice, the results of the first two steps inform the delivery of a web-based PAT Implementation Toolkit.

The key features and contributions of qualitative approaches to engage stakeholders in participatory methods for designing and refining implementation strategies (i.e., gathering process data and explanatory outcome data from which practical ideas can be inductively derived from diverse groups of stakeholders) are well documented [18, 19]. Typically, an implementation science framework is used to facilitate data collection from stakeholders and analyze and interpret their data to identify themes and create or modify implementation strategies [10, 20, 21]. Few research reports detail how stakeholder results informed and transformed initial iterations of implementation strategies, particularly in a long-standing program of research focused on health equity. This paper (a) describes the perspectives of key stakeholders (parent advocates, multidisciplinary health care providers, pediatric oncology provider organization leadership, and leaders in pediatric healthcare industry) about the two strategies for supporting implementation of the PAT and (b) shows how participatory and qualitative methods (semi-structured qualitative interviews with stakeholders) informed and refined the two strategies.

## Methods

### Participants

Stakeholders were selected using purposive criterion-based sampling to represent different levels of the social ecology of children’s cancer including parent advocates, multidisciplinary health care providers, members/leaders of key pediatric psycho-oncology professional organizations, and leaders in the pediatric healthcare policy [20, 21]. Parental stakeholders were considered who had children with cancer. Stakeholders were also sought who had previous experience using the PAT or in delivering other psychosocial screening and intervention approaches. Finally, stakeholders were sought who had expertise in health equity from a firsthand experience, professional practice, and/or healthcare policy. All participants were invited to participate via email. The Children’s Hospital of Philadelphia Institutional Review Board determined that the study was exempt (19-016806[19B0137]). All participants gave verbal informed consent and were offered a $100 preloaded ClinCard to compensate for their time.

### Materials

The semi-structured interview guide used for the interviews was developed by the study team based on ISF, the study framework [15, 22, 23]. Stakeholders were asked to provide in-depth feedback on the two proposed PAT implementation strategies, how to tailor the implementation strategies to sites with different levels of resources, resources needed to implement the PAT, ways to increase family engagement in implementation, communication/translation-oriented issues, and barriers and facilitators to implementation. (See Supplementary Materials- Guide for Implementation Team Stakeholder Interviews and Selecting Barriers and Facilitators to Implementation of the PAT).

### Procedure

Before the interviews, stakeholders were emailed the following materials to familiarize them with the PAT and to review during the interview: PAT in English and Spanish, screenshots of the web-based PAT, graphic of the PPPHM, communication forms for providing families and staff with feedback about screening results, an implementation plan form for sites to outline the details of implementation (i.e., who will screen, when will families be screened, how will results be used), a brief summary of barriers and facilitators to implementing the PAT from past research, and a videoconferencing software guide to minimize technical difficulties.

The electronically recorded interviews (median length=66 minutes) were completed between December 2019 and February 2020 via remote video conference software (BlueJeans) by the principal investigators (A.E.K.; L.P.B.) or the qualitative consultant (J.D.) who provided ongoing oversight of the analysis. Potential conflicts of interest were considered when making the assignments. Professional transcriptions of the electronically recorded qualitative interview were reviewed by R.E.M. to assure accuracy of transcription with input from the interviewers. The transcribed interviews were Microsoft Word documents that were uploaded into Atlas.ti ©. An initial codebook was established with 46 codes, which reflected the interview guide, study questions, and the conceptual perspectives of the study.

To be consistent with the study timeline and rigorously manage a large, complex data set, four coders (R.E.M., G.A.M., K.O., M.A.S.) were selected based on their experience with qualitative research and involvement with projects related to the PAT. All coders received a general orientation to the project by A.E.K and L.P.B. and were trained by J.D. to work in pairs to rigorously and systematically manage, analyze, and interpret qualitative data using the analytic software (Atlas.ti ©) [24–26]. J.D. served as supervisor throughout each phase of the data analysis and conducted training and weekly meetings (February and March 2020).

### Approach to data analysis

**Fig. 2:**
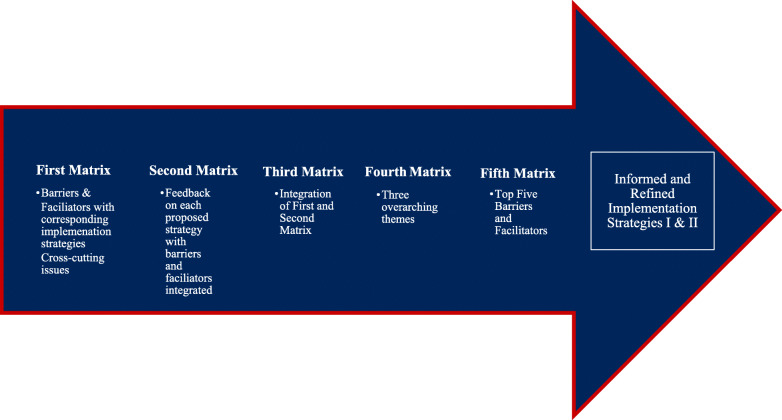
Refining the proposed implementation strategies-feedback to team

Data were analyzed using directed content analysis, which starts deductively with codes based on the study’s conceptual orientation and the ISF, and evolves to include inductively derive codes that accommodate the data [24, 27]. Because of the size and complexity of the interview data set, each pair was composed of a primary and secondary coder who all achieved 75% inter-rater reliability after double coding two-three transcripts. The primary coder then completed the remainder of the assigned transcripts independently while the secondary coder continued to record the data for each stakeholder in a case summary matrix to allow examination of each code, each stakeholder, each type of stakeholder, and all stakeholders. In addition, J.D. and R.E.M. developed a series of five matrices used by the coding pairs to organize and communicate the specific feedback needed to refine the implementation strategies (See Fig. 2). No major modifications were needed to the initial codes, but their descriptions were modified throughout the analysis based on the data collected from the stakeholders. Through discussion among all coders, codes (concrete) were transformed into categories (less concrete and more abstract), and categories were transformed into themes (more abstract statements that fully describe the coding categories) to identify issues and exemplar quotes that stakeholders identified as critical for implementation of the PAT. In addition, the most frequently cited barriers and facilitators were compared among the four stakeholder groups. In weekly meetings over a period of 6 months, coders used the overall themes, summary matrixes, and barriers and facilitators to modify and optimize content and process of the implementation strategies.

The revised implementation strategies were then compared with those in the grant proposal to identify how participatory and qualitative methods specifically informed and refined those strategies. Results of the qualitative analyses, minutes of our meetings, materials prepared for the research proposal, along with the conceptual orientation for the study, were used to assist in the process of identifying the nature of the modifications.

Strategies for strengthening the rigor of the qualitative data analyses were integrated into the study protocol and are reported consistent with the COREQ [25, 26, 28, 29]. Strategies included those integral to strengthening the trustworthiness of the study: integrating the conceptual framework for the study, training and supervision by an expert, gathering data to confirmation study findings, collaborating with team members throughout the analytical process to discern thematic results and to make decisions about refining strategies I and II, and preparing matrices to encourage both inductive and deductive data analyses and interpretation (See Supplementary Materials-COREQ Report).

## Results

### Description of the stakeholders

All stakeholders invited to participate agree to do so. The sample consisted of 19 stakeholders (parent advocates, *n* =5; multidisciplinary health care providers, *n* = 6; pediatric oncology provider organization leadership, *n* = 3; and leaders in pediatric healthcare policy, *n* = 5) who came from 15 organizations and from 11 locales in the USA. All parent advocates had the firsthand experience of having a child with cancer; two were fathers and three were mothers. One parent advocate was a member of a children’s hospital family advisory board. The other four parent advocates are founders of nonprofit organizations that aim to support families affected by childhood cancer. The foundations have a range of perspectives including awareness campaigning, supporting research for psychosocial and medical aspects of pediatric cancer, financially supporting families, and offering peer-support groups for parents of diagnosed children.

The health care providers were clinically experienced social workers, psychologists, and oncologists from programs of various sizes across the USA. Two providers had previous experience implementing the PAT and three providers were at sites in the randomized controlled trial (they were familiar with the PAT but did not directly use the PAT prior to interviews). Provider organization leadership included interviewees who are leaders in the primary professional psychosocial associations, spanning the disciplines of social work, psychology, and nursing. The healthcare policy leaders were from professional institutions that aim to educate, advocate, support research, and define policy and standards to further health equity within their field of expertise. Redundancy (consistency in the interview information) and saturation (no new themes emerging in the interviews) were achieved based on this sample across the stakeholders and within groups of stakeholders throughout the iterative data analysis.

### Stakeholder perspectives

The coders used the overall themes as overarching principles for revising the implementation strategies. Themes for stakeholders as a whole are described as their diverse perspectives were complementary and important in describing how to support implementation of the PAT. Each theme is described with subthemes and exemplar quotes in Table 1.

**Table 1:** Critical issues in implementing screening: themes, sub-themes, quotes, and original codes

Theme	Subtheme	Exemplar quote
Engage providers by framing psychosocial screening as an opportunity for more efficient and effective practice.^a^	Enhances the quality of both medical and psychosocial outcomes	“That’s always the key to the clinical team – is providing the highest quality care – especially if you can do it more efficiently. And I just – I think that’s gotta be part of the messaging to ensure uptake and sustainable uptake.” (Provider)
Set clear expectations about the importance of screening 100% of children and their families to achieve the goal of achieving universal screening, equity of care, and reduction of disparities. ^b^	Becomes normalized as part of care	“So, what are you going to say, some families aren’t deserving of it? No, 100 percent. Are there operational barriers to getting it? Sure. But don’t concede out of the box that you’re not going to get 100 percent.” (Parent)
Adapt successful strategies for systematic implementation of screening to ensure optimal engagement with children and their families throughout their care.^c^	Involves developing/refining capacity for screening (e.g. systems, staff, technical), communication flow within the healthcare team, and capacity for assessing community resources	“You’ve got to have a change in workflow. You’ve really got to have a champion.” (Organization)

^a^Original codes: Strategy I: key points to include are getting the attention of busy clinicians and family-oriented

^b^Original codes: What might be confusing are additional information needed, assure consist use, all families included, equity issues, and tailoring implementation strategies

^c^Original codes: Strategies I and II: results translated into interventions specific to the needs of the family, screening champions, telephone calls, technical support, resources, communication, and translation-oriented

### Stakeholder thematic descriptions

#### Theme 1: Engage providers by framing psychosocial screening as an opportunity for more efficient and effective practice

Stakeholders emphasized the need to acknowledge existing psychosocial risk screening and assessment practices at each site and engage staff at the study sites through explanatory and illustrative accounts of how universal screening with the PAT enhances practice, making it more efficient and effective. Stakeholders described this theme in their general narratives and in their endorsements of specific factors that would facilitate implementation and equity of approach to psychosocial screening (see Table 2). These factors focused on structural or systems changes to the process of patient care (facilitating communication with the family/engaging the family, facilitating communication among staff, facilitating clinical care, assuring no families are excluded) and related outcomes (enhancing quality of care, promoting both medical and psychosocial outcomes, reducing health disparities). Stakeholders from all four groups agreed on the top three facilitators (see Table 2).

**Table 2:** Top five barriers and facilitators across all four stakeholder groups (*n*=19) (based-on barrier and facilitator codes)

Barriers (# of stakeholders)	Facilitators (# of stakeholders)
1. Having staff of services to address identified needs (13)^a^	1. Enhancing quality care to patients and families (11)^a^
2. Communicating results to family (14)^a^	2. Facilitates communication with the family/engages the family (9)^a^
3. Support for the idea of screening from your medical team (8)^a^	3. Promotes positive medical and psychosocial outcomes (7)^a^
4. Time required to use the PAT (6)	4. Facilitates communication among staff (6) Facilitates clinical care (6) Reduces health disparities (6)
5. Identifying a champion (5) Language/cultural considerations (5)	5. Assures no families are excluded (4)

^a^Prioritized in top 3 by all stakeholder groups

First, for strategy 1 (Training), they recommended including the strong evidence base for screening so that participants understood the credibility of the practice. They suggested data-based examples and testimonials to show how screening prioritizes families whose needs are not obvious and are likely to pose significant psychosocial risk requiring clinical services, families whose needs are more subtle yet likely to pose risk needing targeted services, and families who may only need preventive, universal care. Second, they recommended illustrating how screening can change both psychosocial and medical outcomes across the continuum of risk. While psychosocial outcomes are the more obvious outcome, they also suggested using case examples to demonstrate the connection among treatment, psychosocial issues, and medical outcomes (e.g., adherence) that provide added value to the implementation strategy. Third, in terms of strategy II, the champion should not only be familiar with the PAT and its scientific basis but also be able to apply that understanding when advocating for screening in their specific program.

#### Theme 2: Set clear expectations about the importance of screening 100% of children and their families to achieve the goal of universal screening, equity of care, and reduction of disparities

Stakeholders suggested that in order to normalize universal psychosocial screening as part of routine care, both strategy I and strategy II needed to emphasize that all patients on the service or within the selected subgroup should be screened. The specific expectations for the number to be screened each site should be based on the size of the pediatric cancer program and identification of inclusion/exclusion criteria. Stakeholders also explained that flexibility in implementation was needed to allow for innovative problem-solving was required because of differences in patient populations and staff resources. In addition, because the process of patient care is constantly changing due to new treatments and technology and concomitant changes in staff roles and workflow, an approach that is innovatively tailored to the setting is required. They also highlighted that for strategy II, the champion should not only be comfortable with the expectations but be able to provide leadership about how they might be achieved.

#### Theme 3: Adapt successful strategies for systematic implementation of screening to ensure optimal engagement with children and their families throughout their care

In order to successfully implement universal psychosocial screening, the stakeholders recommended awareness of and preparedness to address barriers to implementation. They were cognizant that the process involves developing/refining capacity for screening (e.g., systems, staff, technical), communication flow within the healthcare team, and capacity for accessing community resources. Stakeholders described this theme in their general narratives and in their endorsements of specific factors that could be barriers to implementation (see Table 2). Barriers included more practical issues related to resources (having staff or services to address identified needs, support for the idea of screening from your medical team, time required to use the PAT, identifying a champion) and less issues about using the PAT (communicating the results to families, and language/cultural considerations).

Stakeholders reiterated the need for each children’s program to critically assess capacity to ensure availability of technical resources like electronic tablets. In addition, to fulfill the commitment to universal screening within their specific population, programs might also need to realign staff roles or practices. The flow of communication within the healthcare team was also a concern in that the workflow had to be reconsidered in order to accommodate screening. Finally, community resources and partnerships needed to be considered for the range of needs identified as a consequence of screening.

### Using stakeholder input to modify implementation strategies

A crosswalk which displayed the three qualitative themes, proposed implementation strategies, modifications, their theoretical underpinnings, and their relationships with each other was constructed. As shown in Table 3, participatory and qualitative methods informed the process of refining the two proposed implementation strategies. The table links selected exemplars of modifications made in the two strategies to stakeholder themes and to the conceptual perspectives of the study. Exemplars were chosen because they were clear examples of those links, but do not serve as an exhaustive list of changes made to the two implementation strategies based on these data. The components of the crosswalk were complementary to each other, including their theoretical underpinnings (ISF, PPPHM, social ecology, and health equity). In addition, the matrices summarizing specific suggestions about the content of the webinar and the barriers and facilitators to implementation provided further data-based guidance regarding how to modify the proposed implementation strategies.

**Table 3:** Crosswalk: qualitative themes, proposed implementation strategies, modifications, and links with theory

Stakeholder themes	Proposed strategies	Modifications	Links with theory
Engage providers by framing psychosocial screening as an opportunity for more efficient and effective practice.	Strategy 1^a^ Strategy II^b^	Created a new introduction to the webinar about why screening is important to “you and your patients” that includes parent and professional testimonials Peer consultation calls should not review metrics but concentrate on peer support, problem solving, and best practices	Social ecology Pediatric Psychosocial Preventive Health Model Interactive Systems Framework Prevention Synthesis and Translation (ISF-Phase 1)
Set clear expectations about the importance of screening 100% of children and their families to achieve the goal of universal screening, equity of care, and reduction of disparities.	Strategy 1^a^ Strategy II^b^	Incorporated into webinar; clarity about how to legitimize and normalize use of PAT in your system to be equitable; expectations for screening 100% of population(s) selected; but can define that population for this project to be feasible and achieve success Created job description for the Champion that incorporated recommended elements of the role; reviewed consistency of Implementation Plan regarding Champion and Peer Consultation Calls with related study measures	Health equity ISF-Phase 1
Adapt successful strategies for systematic implementation of screening to ensure optimal engagement with children and their families throughout their care.	Strategy 1^a^ Strategy II^b^	Foreshadowed in beginning of webinar implementation plan development to anticipate and address common barriers and facilitators, access in Spanish, their goal for screening in their setting (e.g. selected population(s); clarify that we will be available during that time to work with them Included in Implementation Plan-Champion’s approach to promote screening at their institution	Social ecology Health equity ISF-Phase 1

^a^Strategy I is training (webinar)

^b^Strategy II is Training + Implementation Expanded Resources (TIER)

Modifications based on the first theme, which centered on the importance of engaging screening staff for both strategies I and II, included creating a new introduction to the training webinar (strategies I and II) covering the reasons that screening is important for both the screening provider and their patients.

Modifications linked to the second theme, which described how the stakeholders thought that both strategies I and II needed to balance expectations and flexibility, included clarity about what is essential (e.g., screening 100% of selected population) and what is inessential (e.g., screening 100% of all patients in all patient populations). Such guidelines were explicitly highlighted as not only important for the process of this project but also because they legitimized and normalized use of the PAT for the outcome of attaining universal screening and equity. Approaches to increase clarity about strategy II expectations included creating complementarity among the job description for the champion, related items on the implementation plan, and study-related measures.

Finally, modifications were made based on the third theme, which identified the importance of developing/refining capacity for screening, communication flow within the healthcare team, and capacity for accessing community resources. The first section of the webinar (strategies I and II) was changed to foreshadow specific components of the PAT implementation plan development which occurs in the last part of the webinar. Specifically, screening staff will be asked to consider common barriers and facilitators, access to the PAT in Spanish, their goal for screening in their setting (e.g., selected population(s) and staff resources. Modifications for strategy II centered on what would be most engaging and supportive in the Consultation calls, such as focusing on peer support, shared problem-solving, and best practices instead of screening metrics as originally planned. An addition was made to strategy II to provide guidance about how the champions can advocate for screening.

## Discussion

While qualitative approaches to engage stakeholders in optimizing implementation strategies are well-documented, few reports detail how stakeholder input is used to do so when strategies are linked to multiple theoretical underpinnings and to long-standing programs of research focused on health equity [10, 16, 18, 19]. This paper makes substantive and methodological contributions to addressing these gaps in the science. First, theoretically informed recommendations based on thematic analysis of stakeholder perspectives revealed that the two proposed implementation strategies should ideally engage the screening providers in the implementation trial, and set expectations for them, as well as enable screening providers to adapt the strategies to their systems of care. These themes contribute insights regarding points to consider when adapting implementation strategies across diverse stakeholders and health care systems. Second, this study demonstrates the outcome of employing rigorous methods based on nationally and internationally informed standards for qualitative research [25, 28, 29] and using content analysis to discover thematic recommendations, matrix analyses to document specific recommendations within and across stakeholder groups, and crosswalks to connect stakeholder themes, proposed strategies, modifications to the strategies, and their theoretical underpinnings. As a whole, these approaches form an innovative bundle of strategies, which efficiently and effectively moved the study forward.

Implementing the PAT, a well-validated psychosocial screening tool that assesses multiple areas of family psychosocial risks and resources related to social determinants of health, in a national sample in pediatric cancer is novel and has the potential to have an important impact on health equity [16]. Building from considerable evidence about acceptability and feasibility of PAT implementation in clinical practice, this project focused on the first component of the implementation process: preparation. Four groups of stakeholders (parent advocates, multidisciplinary health care providers, leaders of key pediatric oncology professional organizations, and leaders in the pediatric healthcare industry) were interviewed to inform and refine proposed implementation strategies. Stakeholders provided valuable feedback to the study team that was used throughout the process of refining the implementation strategies.

Three themes were identified that described their recommendations about the two implementation strategies, including engage providers, provide clear expectations, and adapt successful strategies for systematic implementation of screening. Barriers and facilitators were integrated into those recommendations and reinforced the importance of the broad, theoretically based assessments that are the hallmark of rigorous implementation science [17]. All stakeholders prioritized similar facilitators and barriers; the assessment of facilitators yielded a focus on structural or systems issues and while the assessment of barriers yielded a focus on practical issues and less about actually using the PAT.

Finally, we provided exemplars for how we used those themes and recommendations to modify the implementation strategies. The modifications made in those strategies were not only consistent with stakeholder themes but also to the conceptual orientation of the PAT, PPPHM, and ISF. Findings were interdependent in nature, exposing the importance of capacity building strategies directly related to implementing the PAT that consider and reinforce the importance of an ecological perspective when designing implementation strategies. An example of this was in modification for strategy II in terms of modifications related to health equity. Such modifications (i.e., expecting 100% screening) are consistent with recommended strategies for promoting health equity because they are both practical in that they address an immediate need for individual, communities, and populations and historical structural, systems, and population-based inequities [30].

Because the implementation strategies are being applied to the specific Psychosocial Standard of Care related to screening at diagnosis, their application to other substantive areas of concern across the cancer treatment trajectory or the other Standards are unknown at this time. In addition, the implementation strategies have yet to be tested in the second stage of the ISF (Prevention Support System), thus necessarily limiting the long-term implications for this study which is focused on the important first step of the ISF (Prevention Synthesis and Translation) [15].

Strengths of this study include its strong conceptual basis, stakeholders who were carefully selected to represent diverse stakeholders, solid scientific basis developed over many years of research, and rigorous and transparent data analysis and interpretation. These qualities helped the research team identify meaningful and nuanced outcomes, not only for this phase of the study, but also to build on a substantial knowledge base for the next phase of the study [31]. The study procedures were systematically designed to use data from purposefully sampled stakeholders who had diverse perspectives to make recommendations about the strategies [32, 33]. Their data were compelling and were further amplified through analysis and interpretation using a broad conceptual lens. The approach for this study also balanced the timetable for conducting this preparatory study and for conducting the subsequent cluster randomized implementation trial testing the two implementation strategies. In doing so, the team made effective use of resources and created a coherent plan for moving forward. Based on the first step of the ISF (Prevention Synthesis and Translation System), the study synthesized data to optimize PAT implementation strategies.

## Conclusions

Our results address gaps in the literature related to the content and the process of modifying implementation strategies for family psychosocial screening in pediatric cancer at diagnosis. The value of both practical insights about immediate needs of families as well as historical insights regarding structural inequities such as language differences and access to mental health services to reducing health disparities was realized in gathering and analyzing stakeholder perspectives to modify and optimize the implementation strategies. The resulting strategies address all levels of the social ecology for children’s cancer care, including the patient, family, provider, healthcare system, and community. Further, the methods for deriving qualitative themes, conducting matrix analysis, and performing crosswalks in implementation science are not new, but their configuration and explication within this project are valuable guides for future research. The strong conceptual orientation guiding the methods was important when gathering and analyzing the data and also when integrating results into the two proposed implementation strategies. The qualitative data and study themes helped contextualize the barriers and facilitators for integration into the two proposed implementation strategies in preparation for testing in the PAT implementation trial.

## Supplementary Information

**Additional file 1.** Psychosocial Assessment Tool Implementation Research Network (PAT - IRN): Guide for Implementation Team Stakeholder Interviews

**Additional file 2.** Supplementary Material-COREQ (COnsolidated criteria for REporting Qualitative research) Checklist

**Additional file 3.** Selecting barriers and facilitators to implementation of the PAT -Guide used during interview

## Data Availability

The datasets used and/or analyzed during this study are available from the corresponding author for qualified individuals upon reasonable request.

## Abbreviations

PAT: Psychosocial Assessment Tool
TIER: Training + Implementation Enhanced Resources
ISF: Interactive Systems Framework
PPPHM: Psychosocial Preventive Health Model
ERIC: Expert Recommendations for Implementing Change
