# Supplementary material for: Using qualitative and participatory methods to refine implementation strategies: universal family psychosocial screening in pediatric cancer
Source: Implement Sci Commun. 2021 Jun 5;2:62. doi: 10.1186/s43058-021-00163-4 (PMC8180116; doi:10.1186/s43058-021-00163-4)
Supplement: Supplementary file 1 — Additional file 1. Psychosocial Assessment Tool Implementation Research Network (PAT - IRN): Guide for Implementation Team Stakeholder Interviews [file 43058_2021_163_MOESM1_ESM.doc]

# Supplementary Materials

# Psychosocial Assessment Tool Implementation Research Network (PAT - IRN)

# Guide for Implementation Team Stakeholder Interviews

Materials: Interview Guide (for interviewer only)

The following materials which are sent in advance to the interviewee:

“Paper and pencil” versions of the PAT in English and Spanish

Screenshots of the web-based PAT (three examples)

Pediatric Preventative Psychosocial Health Model (PPPHM)

Communication of Results Form for Families

Communication of Results Form for Staff

PAT Implementation Plan

PAT Implementation Questionnaire Barriers and Facilitators

Equipment: Landline phone (interviewer and interviewee) and computer with internet connection; BlueJeans software; back up audio-recorder for interviewer

Time: Less than one hour (suggested times for each section noted)

Note: This guide is semi-structured in that major topics are presented with potential probes; if new questions or directions arise during the interview they can and should be pursued

**Turn on secondary audio recorder if you are using one.**

1. Welcome (5 minutes)

Thank you for taking the time for this interview. Before we begin, I want to review some logistics about today’s call.

- We are electronically recording the interview using BlueJeans; the electronic file will be transcribed to be sure that we capture what you say as intended. I will also share documents as needed as we proceed via BlueJeans.
- I want to confirm that you have the materials that we sent. We will refer to them as we go.
- Check that the participant has the full packet. Suggest referring to the numbered items in the table of contents as we go:
  - PAT, in English and Spanish (forms) (#1a/b)
  - Screenshot of PAT online (web) (#2)
  - Pediatric Preventative Psychosocial Health Model (#3)
  - Communication of Results Form for Families (#4)
  - Communication of Results Form for Staff (#5)
  - PAT Implementation Plan (#6)
  - Barriers and Facilitators (#7)
- Although we did not plan this part of the study to have a direct benefit to you, many people find interviews like this interesting and learn from them. We do think that we will learn a lot from you and that this will benefit our research.
- You can choose not to continue participation at any time during the interview.
- All information will be confidential; what is discussed in the interview should not be shared with others afterwards. The audiotapes are also confidential. They will not be shared with people outside of our research team and an approved transcription company.
- We will give you a $100 pre-loaded bank card for your participation in this study.
- Do you have any questions about today’s interview? Do you agree to participate?

1. Introduction to the PAT (10 minutes)

I’d like to tell you about the Psychosocial Assessment Tool (PAT) and our plans to increase and evaluate its use in clinical practice in pediatric cancer programs. Our purpose today is to focus on the implementation of the PAT rather than to discuss the content of the PAT. But we did want to be sure that you were familiar with the PAT.

- The Psychosocial Assessment Tool (PAT) is a brief, parent-report, evidence-based family psychosocial risk screener, available in English and Spanish. It is now ready for broad use in clinical practice.
- The PAT is based on a social ecological model and assesses “hot spots” across the child and family’s social ecology. These include assessment of the family’s structure and socioeconomic risks, pre-existing child or family problems, etc. We are particularly interested in aspects of the social ecology that relate to health equity and how early and universal screening can help ensure the delivery of psychosocial care to families to address these risks and reduce disparities.
- Review key elements of the PAT that are relevant to this study:
- Please see the paper and pencil versions of the PAT in English and Spanish (*Handouts #1 and b*)
- The PAT is completed by parents/caregivers online (*Handout #2 for sample screenshots*)
- There are seven subscales, derived from the literature and empirically supported
  - Family Structure and Resources
  - Social Support
  - Stress Reactions
  - Family Problems
  - Child Problems
  - Sibling Problems
  - Family Beliefs
- The total (sum) score maps on to the PPPHM (*show and describe Handout #3*)

Universal Largest group (often about 2/3 of families). Families are stressed but

adaptive and will likely adjust to the cancer diagnosis and treatment

Psychosocial services: General child and family support, family centered care, monitor for changes and problems

Targeted 25-33% of families. Some pre-existing problems.

For Spanish speaking families, there are more at this level

Psychosocial services: Many opportunities for the delivery of evidence based interventions to reduce specific areas of distress and risk

Clinical < 10% of families with increased number of risks

Psychosocial services: Most intense and likely to involve utilization of higher level of resources

- The total score is determined on the PAT after it is completed. Results can be communicated to the family and to health care providers as well as placed in the medical record
  - - Show the Communication of Results to Family Form (*Handout #4*)
    - Show the Scoring Results that go back to staff (*Handout #5*)

1. Implementation of the PAT in clinical practice, a research study (10 minutes)

- We are now studying how to best implement or put the PAT into clinical practice. We need to learn what works best in supporting its use for all families (that is, screening *all* families, not using it only when problems are suspected or already identified). We will test two strategies. Please keep in mind as we go through the interview that we are interested in assuring that screening is universal and identifies families, both English and Spanish speaking, at risk for health disparities. In this study, we plan to compare two strategies for implementation.
- Strategy I uses education about the PAT in general and how to use it. The training will be a webinar and will include a description of the PAT, information about the web-based PAT and how to access and use it, and information about understanding what the scores mean and how to provide feedback to families about their scores. As part of the training, each site will develop a site-specific PAT Implementation Plan (*refer to the PAT Implementation Plan in Handout #6* and review briefly). The protocol will specify who does screening, who is screened, how screening is accomplished, where the information will go and how it will be used. We will provide basic technical assistance to sites to support their use.
  - What do you think of this implementation strategy?
  - What are the key points that we should include in the PAT Webinar?
  - What might be confusing to clinicians about the PAT and its use?
  - What can we do to keep the attention of busy clinicians and make the time viewing the PAT Webinar “worth their while”?
  - What additional information do you think is important to include in the PAT Implementation Plan to assure that the PAT gets put into practice and used properly?
  - What information would be helpful in terms of using the PAT in Spanish?
  - How can we assure through our webinar training content that staff are consistenly screening all families, including those with many stressors or those more disengaged from the healthcare system?
  - To what extent is technical support for staff or families integral to PAT implementation?
- Strategy II includes the same PAT Webinar and adds “Peer Support” by providing regular guidance through “consultation calls” with sites as a group to create a Learning Collaborative. In addition, each site will designat a “Screening Champion” (someone who advocates for and supports screening with the PAT).
  - What do you think of this implementation strategy?
  - What kinds of topics and support would be most helpful to the sites during the calls?
  - Which roles in healthcare might serve as strong screening champions?
  - What are three things that a champion could do to help assure successful implementation?
  - What other ideas do you have in terms of how to support staff in screening or components we might add beyond the PAT Webinar?

1. Barriers and Facilitators that may arise in implementation through either Strategy I or II (10 minutes)

- We have identified barriers and facilitators to screening that we anticipate will be important to recognize and address in implementing the PAT.
  - Show a list (*Handout #7*) and review very briefly.

- Thinking about our strategies, please pick three barriers that you believe are most important or relevant to limiting successful, universal family psychosocial risk screening at diagnosis. For each of the three:
  - How is this a barrier? Who is it a barrier for (families, providers, institution)?
  - Does this barrier affect certain patients, families, staff, or institutions more than others?
  - How can we address this barrier through the implementation strategies we evaluate? In the Webinar? Through peer support?
- Now please pick three facilitators of screening that you believe are the most important or relevant to successful, universal family psychosocial risk screening at diagnosis. For each of the three:
  - How is this a facilitator? Who is it a facilitator for (families, providers, institution)?
  - Does this facilitator affect certain patients, families, staff, or institutions more than others?
  - How can we address this facilitator through the implementation strategies we evaluate? In the Webinar? Through peer support?

1. Additional Questions about PAT Implementation and Health Disparities (20 minutes)

*Some of these topics will likely have been addressed in the prior questions. Pick questions that will further expand the discussion and ideas for implementation*. *We have highlighted questions to priorize at this point if they have not already been discussed.*

- Tailoring-oriented
  - *To what extent do we need to tailor implementation strategies to different size sites in terms of number of new patients per year or sites with differing types of structures or psychosocial staffing?*
- Resource-oriented
  - *As we think about implementing the PAT, what resources would be necessary to use the PAT in a systematic manner? Probe for necessary resources generally and why.*
  - If we provide healthcare providers/systems with a “toolkit” for PAT implementation, what information is most helpful in terms of implementation? What are the three most important things to be sure that the toolkit contains?
  - Please comment on your experiences with peer support models (in this study consultation calls) and how they may help or hinder implementation.
  - *How can representatives of insurance companies, advocacy groups, or others outside the immediate healthcare system (or children’s cancer centers) be involved in supporting the implementation of screening? How might the impact of family psychosocial risk screening on health care costs be communicated to these organizations to promote investment in screening?*
- Family-oriented
  - How can families be involved in the process of implementing screening?
  - *What issues should be considered for all families, including those from racial or ethnic minority backgrounds and/or from socioeconomically challenged situations?*
  - *How about families who speak Spanish? What considerations should we consider for these families?*
- Communication and translation-oriented
  - *Assuring that key clinical data is incorporated into electronic health records is critical. How might we take steps to assure that the information is made available to all parties (families, providers)?*
  - *How do we take the results of screening and translate them into interventions specific to the needs of families? This is the site-specific blueprint for care or PAT Implementation Plan. Please draw upon your experiences and think creatively about ways to assure that evidence-based care is the ultimate outcome of screening.*
  - *Thinking “outside the box” for a minute, what else might we consider as we implement the PAT? Please be broad in your thoughts about this.*

1. Thanks and conclusion (5 minutes)

*The goal of this section is to thank participants and summarize the discussion with general ideas about their input.*

Our plan is that your input today will be used to help us better understand how the PAT could be integrated into practice to match psychosocial care to psychosocial risk, to reduce disparities in care, decrease distress, and improve health-related quality of life for children with cancer and their families. We will integrate the information from across interviews and refine Strategy I and Strategy II accordingly. Thanks again for your commitment to our research and for your time today. Please feel free to contact us via email or phone with any additional thoughts.
