# Supplementary material for: Using qualitative and participatory methods to refine implementation strategies: universal family psychosocial screening in pediatric cancer
Source: Implement Sci Commun. 2021 Jun 5;2:62. doi: 10.1186/s43058-021-00163-4 (PMC8180116; doi:10.1186/s43058-021-00163-4)
Supplement: Supplementary file 3 — Additional file 3. Selecting barriers and facilitators to implementation of the PAT -Guide used during interview [file 43058_2021_163_MOESM3_ESM.docx]

Supplementary Materials

Selecting barriers and facilitators to implementation of the PAT -Guide used during interview

Barriers/Challenges

1. Support for the idea of screening from your medical team

2. Identifying a champion(s)

3. Time required to use the PAT

4. Family willingness to complete PAT

5. Reimbursement for screening

6. Finding a staff member to conduct screening

7. Technical issues with forms/electronic materials

8. Responding promptly and appropriately to urgent needs (“red flags”)

9. Communicating results to families

10. Communicating results to staff

11. Integrating screening results in the EHR

12. Having staff or services to address identified needs

13. Language barriers/Cultural considerations

Facilitators/Benefits

1. Facilitates communication among staff

2. Facilitates communication with the family / engages the family

3. Facilitates clinical care

4. Enhancing quality care to patients and family

5. Reduces health disparities

6. Efficient and effective model of care delivery

7. Standardizes how sensitive issues are raised

8. Reduces likelihood of unnecessary referrals and costs

9. Promotes positive medical and psychosocial outcomes

10. Assures no families are excluded
